# The GPCR adaptor protein Norbin regulates S1PR1 trafficking and the morphology, cell cycle and survival of PC12 cells

**DOI:** 10.1038/s41598-023-45148-6

**Published:** 2023-10-25

**Authors:** Valdemar B. I. Johansen, Elizabeth Hampson, Elpida Tsonou, Chiara Pantarelli, Julia Y. Chu, Laraine Crossland, Hanneke Okkenhaug, Andrew J. Massey, David C. Hornigold, Heidi C. E. Welch, Stephen A. Chetwynd

**Affiliations:** 1 Signalling Programme, The Babraham Institute, Babraham Research Campus, Cambridge, CB22 3AT UK; 2 Department of Biology, Faculty of Science, University of Copenhagen, Copenhagen, Denmark; 3 Faculty of Health and Medical Sciences, University of Copenhagen, Copenhagen, Denmark; 4 Vernalis (R&D) Ltd., Cambridge, UK; 5 Bioscience Metabolism, Research and Early Development, Cardiovascular, Renal and Metabolism (CVRM), BioPharmaceuticals R&D, AstraZeneca, Cambridge, UK; 6 Imaging Facility, The Babraham Institute, Cambridge, UK

**Keywords:** Cell biology, Neuroscience, Physiology

## Abstract

Norbin is an adaptor protein that binds numerous G protein-coupled receptors (GPCRs), is highly expressed in neurons, and is essential for a functioning nervous system in rodent models. Yet, beyond its control of neurite outgrowth and synaptic plasticity, few cellular roles of Norbin have been investigated to date. Furthermore, while Norbin is known to regulate the steady-state cell surface levels of several GPCRs, only in one case has the protein been shown to control the agonist-induced receptor internalisation which serves to attenuate GPCR signalling. Here, we generated a Norbin-deficient PC12 cell line which enabled us to study both the cellular functions of Norbin and its roles in GPCR trafficking and signalling. We show that Norbin limits cell size and spreading, and is required for the growth, viability and cell cycle progression of PC12 cells. We also found that Norbin regulates both the steady-state surface level and agonist-induced internalisation of the GPCR sphingosine-1-phosphate receptor 1 (S1PR1) in these cells, suggesting that its role in agonist-dependent GPCR trafficking is more widespread than previously appreciated. Finally, we show that Norbin limits the S1P-stimulated activation of Akt and p38 Mapk, and is required for the activation of Erk in PC12 cells. Together, our findings provide a better understanding of the cellular functions of Norbin and its control of GPCR trafficking.

## Introduction

Norbin (Neurochondrin, Ncdn) is an essential, yet understudied, 79 kDa adaptor protein^1–5^. It is highly conserved among vertebrates, without any catalytic activity or sequence homology to other proteins, and is predicted to consist almost entirely of HEAT repeats^1,2,4,6,7^. General Norbin deficiency in mice is early embryonic lethal^4^, whereas mice with targeted Norbin deletion in the nervous system present with epilepsy^8^, those with Norbin deficiency in the postnatal forebrain exhibit schizophrenic behaviours^5^, and those with hippocampal Norbin deficiency show depressive-like behaviours^9^. It seems likely that these murine phenotypes are relevant to humans, as downregulation of Norbin expression is commonly seen in patients with schizophrenia and epilepsy^10,11^. A recent study identified six patients displaying varying degrees of developmental delay, intellectual disability, and epilepsy, with four separate inherited or de novo missense mutations^12^. In contrast to wild-type Norbin, variants carrying either of these mutations were unable to restore neurite-outgrowth in Norbin-deficient neuroblastoma cells^12^. Another recent study identified a de novo nonsense mutation in a patient with frontotemporal dementia, corresponding to reduced Norbin expression in the brain and suspected functional haploinsufficiency^13^. Furthermore, Norbin has been identified as the neuronal target of auto-antibodies in autoimmune cerebellar ataxia^14,15^ and chorea minor^16^.

Norbin was identified as a cytoplasmic protein in neurons, where it is highly expressed and required for neurite outgrowth and synaptic plasticity^2,5,6,12,16,17^. How Norbin regulates these neuronal responses, and if it controls others, is currently unknown. Recent work from our lab showed that Norbin is not only expressed in neurons, but also in myeloid cells, where it functions as a negative regulator of innate immunity^18,19^. Mice with myeloid Norbin deficiency have elevated immunity to bacterial infections, due to increased neutrophil responsiveness^19^. Norbin-deficient neutrophils show an altered morphology, constitutively spreading more than wild type neutrophils during adhesion. They also kill more bacteria, degranulate and phagocytose better, and produce more reactive oxygen species and neutrophil extracellular traps than wild type neutrophils in response to a range of stimuli, but especially upon stimulation of G protein-coupled receptors (GPCRs)^19^.

Norbin binds directly and constitutively to a large number of GPCRs (35 GPCRs out of 55 tested to date), for example the class A melanin-concentrating hormone receptor 1 (MCHR1) and the class C metabotropic glutamate receptor 5 (mGluR5)^5,20,21^. It binds to the intracellular C-terminal tail of the GPCRs through its own C-terminus, and can regulate the steady-state cell surface levels of those GPCRs that it interacts with directly^5,21,22^. Unfortunately, primary GPCR sequence is insufficient to predict which GPCRs Norbin can bind^5,20^, so interactions need to be determined empirically. We recently demonstrated constitutively raised cell-surface levels of C5aR1 and CXCR4 in Norbin-deficient neutrophils, suggesting that these GPCRs may be new Norbin targets^19^. While Norbin seems to regulate the steady-state surface levels of GPCRs quite widely, it was thought that Norbin may not be involved in the homologous desensitisation of GPCRs by clathrin-mediated endocytosis. This was based on a study on MCHR1, where Norbin expression did not affect agonist-induced internalisation^21^. However, recently Norbin was shown to regulate the agonist-induced internalisation of mGluR5, as well as the constitutive cell-surface level of this receptor^22^. Norbin is also known to regulate the signalling of GPCRs that it interacts with directly^1^. GPCR signalling pathways affected by Norbin include Erk, Rac and Ca^2+^, and it can regulate these pathways up or down, depending on the GPCR and cellular context^1^. Overall, the functional significance of the majority of Norbin/GPCR interactions, and the mechanisms through which Norbin regulates GPCR trafficking and signalling, remain elusive.

Sphingosine-1-phosphate receptors (S1PRs) are a group of five GPCRs activated by sphingosine 1-phosphate (S1P), a lipid-signalling mediator that is essential for neuronal function^23^. Deregulation of S1PR signalling leads to neurodegenerative and neuro-inflammatory diseases^23^. Among the five S1PRs, the Gα_i_-coupled S1PR1 is the most highly expressed receptor in the nervous system^24^, and is required for the proliferation, migration and differentiation of neuronal stem cells^25,26^. Norbin is known to bind S1PR1 directly^20^. However, the significance of this interaction is currently unknown.

Cellular functions of Norbin known today include neurite outgrowth and synaptic plasticity in neurons^1,2,5,6,17^, and the host defence responses we identified in neutrophils^19^. These are all responses of postmitotic, differentiating or fully differentiated cells. A role in proliferation was proposed in adult neurogenesis, when decreased BrdU incorporation was seen in mice with nervous system-specific Norbin deletion, however this was concluded to be a non-cell autonomous effect^9^. We sought to investigate here if Norbin may fulfil any previously unidentified roles in proliferating cells. In addition, we were intrigued by a recent finding that Norbin may not only regulate the steady-state surface levels of GPCRs but also their agonist-induced internalisation^22^. Hence, we decided to study cellular functions and GPCR trafficking roles of Norbin in PC12-S1PR1 cells.

PC12 cells were originally derived from a rat pheochromocytoma, and are one of the most commonly used cell lines in neuroscience research. They express several neuronal receptors, are capable of neurotransmitter secretion, morphologically resemble neurons upon differentiation with nerve-growth factor (NGF), and are amenable to experimental manipulation^27^. PC12-S1PR1 is a PC12 cell line that we recently established, which stably expresses S1PR1 with a C-terminal GFP tag, at a level approximately 3% over the abundant endogenous S1PR1^28^. Here, we generated Norbin-deficient PC12-S1PR1 cells by CRISPR/Cas9-mediated knockout to compare cell responses, as well as S1PR1 trafficking and signalling, between wild type and Norbin-deficient PC12-S1PR1 cells. We show that Norbin controls the size, morphology, growth, survival and cell-cycle progression of PC12-S1PR1 cells, and that it regulates both the steady-state surface level and the S1P-induced internalisation of S1PR1, as well as S1P-stimulated signalling through Akt, p38 Mapk, and Erk. Thus, our study provides novel insight into functional roles of Norbin in cell responses and in GPCR trafficking and signalling.

## Materials and methods

### Cell culture

PC12 rat phaeochromocytoma cells were a kind gift from Dr Llewelyn Roderick, Babraham Institute, Cambridge, UK. From these, we derived PC12-S1PR1 cells which stably express C-terminally GFP-tagged S1PR1, as described previously^28^. Cells were maintained in complete Dulbecco’s Modified Eagle’s Medium (DMEM, Gibco, 41,965–039), supplemented with 10% horse serum, 5% foetal bovine serum, 1 U/ml penicillin and 1 U/ml streptomycin (Gibco, 15,140–122), 2 mM glutamine, and 500 µg/ml G-418 disulphate (Melford, Ipswich, UK, G0175)) in poly-D-lysine coated vented tissue culture flasks (Nunc) at 37 °C in a humidified, 5% CO_2_ incubator. The flasks were coated with poly-D-lysine for 1 h at RT and washed twice in tissue culture-grade water (Hyclone, SH30529.02) prior to use. Wild type and Norbin-deficient PC12-S1PR1 cells, generated as described here-below, were passaged by trypsinisation with either 0.25% trypsin–EDTA (Thermo Fisher Scientific, Loughborough, UK, 25,200,056) or TrypLE Express (Thermo Fisher Scientific, 12,604,013) and were used in experiments at between 1 and 7 weeks in culture. For experiments, cells were plated in tissue culture plates 1–3 days prior to the experiment so that they were 50–70% confluent on use, as specified. For cryopreservation, cells were frozen in FBS, 10% DMSO (Sigma, 276,855) at – 80 °C overnight and then stored in liquid nitrogen.

### Generation of Norbin-deficient PC12-S1PR1 cells

Norbin-deficient PC12-S1PR1 cells were generated by CRISPR-Cas9 mediated knockout. Three sgRNAs were designed using design software developed by the Feng Zhang laboratory (http://crispr.mit.edu/), to match the following criteria: to direct Cas9 nuclease-mediated double-strand cleavage to exons 3 or 4 of the rat Ncdn genomic sequence, to be positioned directly upstream of a requisite 5′-NGG protospacer adjacent motif (PAM), and to have no homologous sites elsewhere in the rat genome. The sgRNAs were obtained from Sigma Aldrich (Haverhill, UK) and validated in vitro using the Guide-it sgRNA In Vitro Transcription and Screening kit (TakaraBio, Saint-Germain-en-Laye, France, 632636). sgRNA AAAGATCCTACGTCGAGTTTTGG, which targets exon 3, was selected as the most efficacious. PC12-S1PR1 cells were transfected with this sgRNA and with a GeneArt CRISPR Nuclease Vector that expresses orange fluorescent protein (OFP) (Thermo Fisher Scientific, A21174) using JetPEI transfection reagent (Polyplus 101-10N), following the manufacturer’s protocol. 44 h later, the cells were resuspended in DMEM, 1% FBS, 2 mM glutamine, and OFP-positive cells were enriched by fluorescence-activated cell sorting as described^28^. Single cells were sorted into 96-well plates and cultured for clonal expansion in the complete growth medium. 32 clonal cell lines were established. Clones were screened by extracting genomic DNA, amplifying the region of interest by PCR, with primers GAAGGTGGCGGAAATGGATC and GCATAGCAGGCAGTTACCAC, and analysing the resulting DNA fragments by Sanger sequencing (GenBank accession number: OQ559938). To confirm the Norbin-deficiency on the protein level, total cell lysates were prepared and western blotted using Norbin C1 antibody, as previously described^18^. Total protein loading was tested by Coomassie blue staining after blotting. Among the 32 clonal cell lines obtained, Norbin was found to be successfully deleted in one single cell line. This Norbin-deficient PC12-S1PR1 cell line was compared to wild-type PC12-S1PR1 cells throughout the study.

### Cell morphology

Wild type and Norbin-deficient PC12-S1PR1 cells were seeded onto 13 mm glass coverslips (Thermo Fisher Scientific, 12392128) in 24-well plates (Nunc, 142475) and grown to 50–60% confluence. Cells were serum-starved overnight in DMEM, 0.1% fatty-acid free BSA (Sigma, A8806). The starved cells were fixed with 4% paraformaldehyde (PFA), 50 mM Pipes (pH 6.5), 1 mM EGTA, 10 mM MgCl_2_ for 15 min at RT, washed three times in DPBS, stained with Hoechst 33342 DNA dye, and mounted onto microscope slides using Aqua-Poly/Mount (Polysciences, 18606–20). Cells were imaged using a Nikon AR1 confocal microscope (60 × objective) and images analysed in a blinded manner using ImageJ. Images were adjusted for contrast and brightness, and a threshold of the S1PR1-GFP signal was set to generate a mask for each cell. The Set Measurements function of ImageJ was used to determine cell area and perimeter, with particles smaller than 40 µm^2^ being excluded from the analysis. Circularity was calculated by ImageJ as 4π(area/perimeter^2^). As an additional evaluation of cell morphology, we compared the forward and side scatter characteristics of wild type and Norbin-deficient PC12-S1PR1 cells by flow cytometry, as described below.

### Cell growth, viability and cell cycle

Cell growth, viability, and cell cycle stages were assessed essentially as described^28^. Briefly, wild type and Norbin-deficient PC12-S1PR1 cells were seeded into poly-D-lysine coated 12-well tissue culture plates (Nunc, 150628) at a density of 2 × 10^4^ cells per well. Once every 12 or 24 h, for 7 days, cell growth and viability were assessed by cell counting and exclusion of 0.4% trypan blue dye using a haemocytometer. The medium was changed once on day 3. In addition, viability was assessed by flow cytometry, in cells grown for two days to 60% confluence, by using exclusion of fixable viability dye to identify live cells, as described below for the GPCR internalisation assay. To examine cell cycle stages, 3 × 10^5^ cells were seeded into a 6-well plate, harvested two days later by scraping, sedimented at 10^4^×*g* for 30 s at 4 °C, and washed twice in DPBS. Cells were fixed in 70% ice-cold ethanol for 30 min on ice, washed twice with DPBS, and incubated with 5 µg/ml DAPI for 30 min on ice. Samples were analysed by flow cytometry on a BD LSR Fortessa Cell Analyzer, recording a minimum of 10^4^ events for forward scatter (FSC), side scatter (SSC), and DAPI staining using a 355 nm UV laser and 450/50 bypass emission filter. The gating strategy is shown in Supplemental Fig. 1A. The Watson model on FlowJo (10.6.1) was used to assign cells to sub-G_1_, G_1_, S, and G_2_/M cell cycle stages according to their DAPI content.

### GPCR internalisation (imaging)

To measure the agonist-stimulated internalisation of S1PR1-GFP by imaging, wild type and Norbin-deficient PC12-S1PR1 cells were plated and serum-starved as described above for the morphology assay, and were then stimulated with increasing concentrations of S1P (Sigma, S9666), as indicated, for 10 min at 37 °C, prior to fixing with 4% PFA, and were imaged using confocal microscopy as detailed above. Images were analysed using CellProfiler. A mask covering the entire cell was generated. This mask was then shrunk by 9 pixels, the mean depth of the plasma membrane, to create a second mask representing the cytoplasm and nucleus. Subtracting the second mask from the first gave the plasma membrane compartment, and subtracting the nuclear DAPI stain from the second mask gave the cytoplasmic compartment. The mean GFP intensities in these compartments were used to quantify the relative abundance of S1PR1-GFP at the plasma membrane.

### GPCR internalisation (flow cytometry)

To quantitate the cell surface level of S1PR1 and the agonist-induced internalisation of the receptor, we used flow cytometry. Wild-type and Norbin-deficient PC12-S1PR1 cells were seeded into 6- or 12-well plates, grown to 60% confluence, and starved overnight in serum-free medium, prior to stimulation with the indicated doses of S1P (or mock stimulation) for 30 min at 37 °C. Plates were transferred onto metal trays on ice. Cells were harvested by scraping, centrifuged at 10^4^×*g* for 30 s at 4 °C, resuspended in 0.1% Fc block (BD Biosciences, 553141) in DPBS (Thermo Fisher Scientific, 14040117) supplemented with 0.1% glucose and 4 mM NaHCO_3_ (DPBS^++^), and incubated for 15 min on ice. An aliquot of cells was heat-killed for 10 min at 55 °C, cooled, and then pooled 50/50% with an aliquot of live cells, for use as a viability control. After the incubation in Fc block, cells were pelleted at 10^4^×*g* for 30 s at 4 °C, resuspended in DPBS^++^ containing 0.1% Fc block and 4 µg/ml mouse anti-S1PR1 antibody (R&D Systems, MAB2016), and incubated for 30 min on ice. Cells were washed twice in DPBS^++^, resuspended in DPBS^++^ containing 0.1% Fc block, 0.1% eFluor 780 fixable viability dye (Thermo Fisher Scientific, 65086514), and 4 µg/ml AF568-labelled goat anti-mouse IgG (Thermo Fisher Scientific, 11004), and were incubated for 30 min on ice. Cells were washed twice in DPBS^++^ and resuspended in Hank’s Balanced Salt Solution (Sigma, H6648) with 15 mM HEPES, pH 7.4 (Sigma, H3784), 0.25% fatty acid-free BSA (Sigma, A8806) and 1 mM EDTA. Flow cytometry was performed on a BD LSRFortessa™ Cell Analyzer, recording FSC, SSC and fluorochromes. GFP was excited using the 488 nm laser and recorded with a 530/30 nm bypass filter. AF568-labelled goat-anti mouse IgG was excited with a 561 nm laser and recorded using a 585/15 bypass filter. The eFluor 780 fixable viability dye was excited using a 640 nm laser and recorded with a 780/60 bypass filter. The gating strategy is shown in Supplemental Fig. 1. Samples were analysed using FlowJo for the cell surface level of S1PR1. To determine the total amount of S1PR1, cells were fixed in 4% PFA for 15 min at RT, washed in DPBS^++^, permeabilised in DPBS^++^, 0.5% Tween-20 for 10 min at RT, and washed again, prior to blocking in DPBS^++^, 0.1% Fc block for 30 min, staining with the mouse anti-S1PR1 antibody for 1 h and AF568 anti-mouse IgG secondary antibody for 30 min, and analysis by flow cytometry as described here-above.

### S1P signalling

S1P signalling was evaluated by western blotting, essentially as previously described^28^. Briefly, wild-type and Norbin-deficient PC12-S1PR1 cells were seeded into poly-D-lysine coated 6-well culture plates at 3 × 10^5^ cells per well. The next day, they were serum-starved in DMEM containing 0.1% fatty acid-free bovine serum albumin for 16 h prior to stimulation with 3 × (150 nM) S1P to give a final concentration of 50 nM, for the indicated periods of time at 37 °C. The medium was aspirated, plates were placed onto an iced metal tray, and cells were lysed in 200 μl ice-cold RIPA buffer supplemented with 2 mM DTT, 0.1 mM PMSF, and 10 μg/ml each of leupeptin, antipain, aprotinin, and pepstatin-A, for 5 min on ice. Samples were transferred into precooled Eppendorf tubes, insoluble material was removed by centrifugation at 12,000×*g* for 5 min at 4 °C, and the supernatant (total lysate) transferred into fresh precooled tubes. Proteins were denatured by the addition of 70 μl boiling 4 × SDS sample buffer to give 1.3 × final, resolved by SDS-PAGE, and Western blotted. Primary antibodies, from Cell Signaling Technology (London, UK), comprised phospho-Akt S473 (9271, 1:500), Akt (9272, 1:1000), phospho-p38 Mapk (9211, 1:300), p38 Mapk (9212, 1:1000), phospho-Erk1/2 (4370, 1:2000), Erk1/2 (9102, 1:1000). Secondary antibodies were HRP-conjugated goat anti-mouse IgG (Bio-Rad, Watford, UK, 1706516) or goat anti-rabbit IgG (Bio-Rad, 1706515, 1:3000). Detection was done using Clarity ECL reagent (Bio-Rad, 1705060, 1:3000). Blots for phosphorylated proteins were done first, then stripped, and reprobed for the total proteins. The activities of Akt, p38 Mapk and p44/42 Erk were quantified using ImageJ densitometry, and the phospho-signals divided by the total for each protein.

### Experimental design and statistical analysis

GraphPad Prism 9 (GraphPad Software, San Diego, CA, USA) was used for tabulation, statistical analysis and plotting graphs. Data were tested for normality of distribution to determine if parametric or non-parametric statistical analysis was required. Where warranted by variance between groups, data were log-transformed prior to statistical analysis. Statistical outliers were identified using ROUT test and removed from datasets. Other samples were only excluded when there was a known technical problem affecting the analysis. For comparisons between two groups, two-tailed Student’s t-test was used. For testing the effects of interventions, two-way ANOVA with Sidak’s post-hoc multiple comparisons corrections test was used. Effect size and variance are reported as group mean ± standard error. The threshold for statistical significance was set at *p* < 0.05. Where relevant, non-significant p-values are indicated in grey in the figures. Group sizes and numbers of experimental repeats are listed in the figure legends.

## Results

### Generation of Norbin-deficient PC12-S1PR1 cells

**Figure 1:**
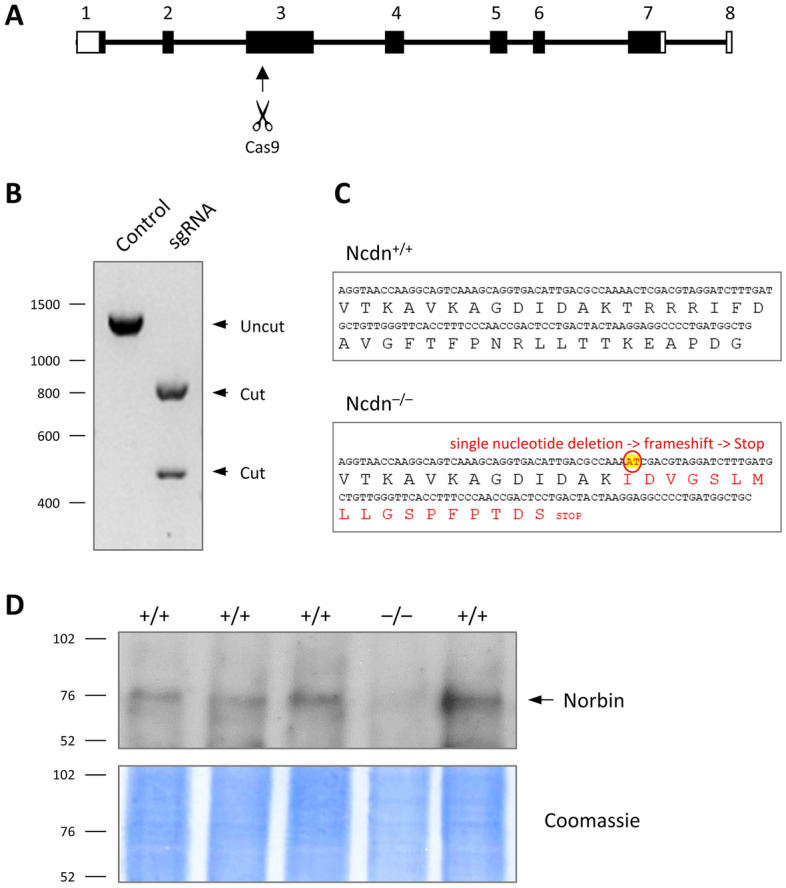
Generation of Norbin-deficient PC12-S1PR1 cells. (**A**) Exon–intron structure of the rat Norbin gene, Ncdn. The arrow denotes the targeting site in exon 3 chosen for generating Norbin-deficient PC12-S1PR1 cells by CRISPR/Cas9 knockout. (**B**) In vitro testing of the sgRNA selected for CRISPR/Cas9-mediated targeting of Norbin. The gel shows cutting of the Ncdn exon 3 target DNA by Cas9 in the presence of the sgRNA. (**C**) DNA and amino-acid sequence of the region of interest within exon 3 of rat Ncdn in wild-type (Ncdn^+/+^) and Norbin-deficient (Ncdn^–/–^) PC12-S1PR1 cell clones. CRISPR/Cas9 gene editing caused homozygous deletion of a single nucleotide in exon 3 of one clone, resulting in frameshift and premature Stop. (**D**) Western blot analysis of wild-type (^+/+^) and Norbin-deficient (^–/–^) PC12-S1PR1 cell clones for Norbin expression; coomassie staining was used to control for protein loading.

To study the role of Norbin in cell responses and GPCR trafficking, we used PC12-S1PR1 cells, which are PC12 cells that stably express the GPCR S1PR1 with a C-terminal GFP tag^28^. We deleted Norbin from these cells by CRISPR/Cas9 knockout. For this, sgRNAs were designed to target the Norbin gene, Ncdn, and were tested in vitro for efficacy. The most efficient sgRNA, directed against exon 3, was selected to guide Cas9 nuclease to inducing double-strand breaks in the target sequence (Fig. 1A–B). Clonal cell lines were generated and assessed for Norbin deficiency by both DNA sequencing and Western blotting. Sequencing identified one homozygous cell clone in which the gene editing had successfully deleted a single nucleotide from exon 3, causing frameshift, premature Stop, and Norbin knockout (Fig. 1C). Western blotting confirmed the deletion of Norbin protein from this Ncdn^–/–^ cell line (Fig. 1D). The Norbin-deficient PC12-S1PR1 cell line was compared to wild type (Ncdn^+/+^) PC12-S1PR1 cells throughout this study.

### Norbin regulates the morphology of PC12 cells

**Figure 2:**
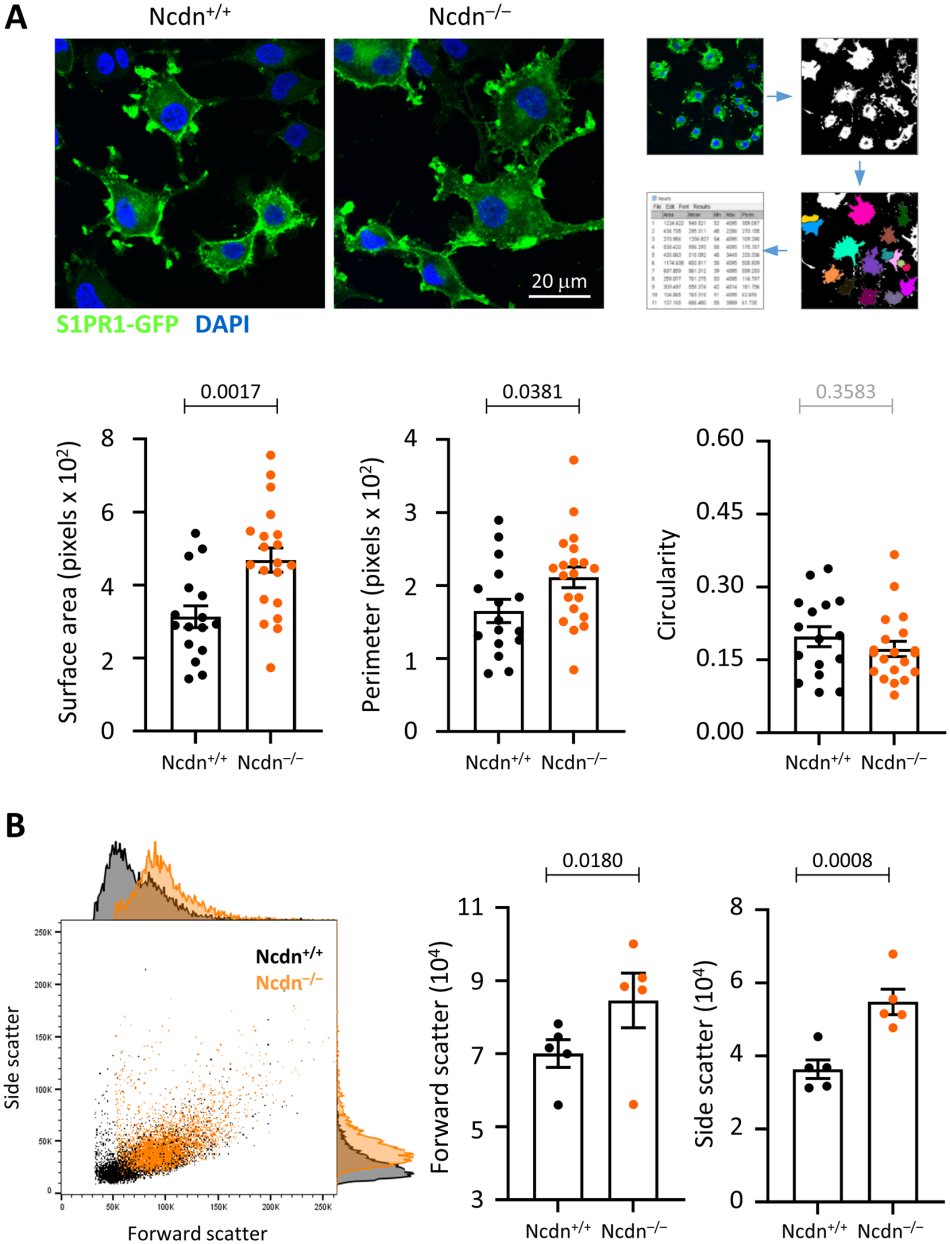
Norbin regulates the size and morphology of PC12-S1PR1 cells. (**A**) Imaging of Norbin-deficient PC12-S1PR1 cells. Top panels: Confocal fluorescence microscopy images of serum-starved wild type (Ncdn^+/+^) and Norbin-deficient (Ncdn^–/–^) PC12-S1PR1 cells from one experiment representative of three. The cells express S1PR1-GFP (green) and were stained with DAPI DNA dye (blue). Right-hand panel: schematic of ImageJ analysis. A mask was generated for each cell and analysed for cell surface area, perimeter and circularity. Bottom panels: Quantification of the morphology of serum-starved Ncdn^+/+^ (black) and Ncdn^–/–^ (orange) PC12-S1PR1 cells by ImageJ analysis. Data are mean ± SEM of cells in 16 fields of view (fov) for Ncdn^+/+^ and 20 fov for Ncdn^–/–^, pooled from three independent experiments; statistics are unpaired t-test. (**B**) Flow cytometric analysis of serum-starved Ncdn^+/+^ (black) and Ncdn^–/–^ (orange) PC12-S1PR1 cells. Left-hand panel: representative flow cytometry plot from one experiment representative of five, with histograms for forward (FSC-A) and side scatter (SSC-A) characteristics. Middle and right-hand panels: Quantification of FSC and SSC. Data are mean ± SEM of five independent experiments; statistics are paired t-test.

We used confocal imaging of wild type and Norbin-deficient PC12-S1PR1 cells to assess if Norbin deficiency affects cell morphology. Indeed, serum-starved Norbin-deficient PC12-S1PR1 cells appeared larger and more spread than wild type cells (Fig. 2A). Quantification by ImageJ analysis confirmed that the Norbin-deficient PC12-S1PR1 cells did have both a significantly larger surface area and perimeter than wild type PC12-S1PR1 cells (Fig. 2A). In contrast, the circularity of the cells was normal, indicating that the increases in cell area and perimeter were proportional, and therefore that the relative amount of membrane protrusions was not affected by the Norbin deficiency, at least under the serum-starved condition tested (Fig. 2A). Similarly, Norbin-deficient PC12-S1PR1 cells were also found to be larger than wild type when analysed by flow cytometry for their light scattering characteristics (Fig. 2B; see Supplemental Fig. 1 for the gating strategy). Together, these data show that Norbin limits the size and spreading of undifferentiated, proliferating PC12 cells. This adds to a previous paper which showed that Norbin controls neurite outgrowth in PC12 cells upon differentiation with NGF^17^.

### Norbin regulates the growth, viability and cell-cycle progression of PC12 cells

**Figure 3:**
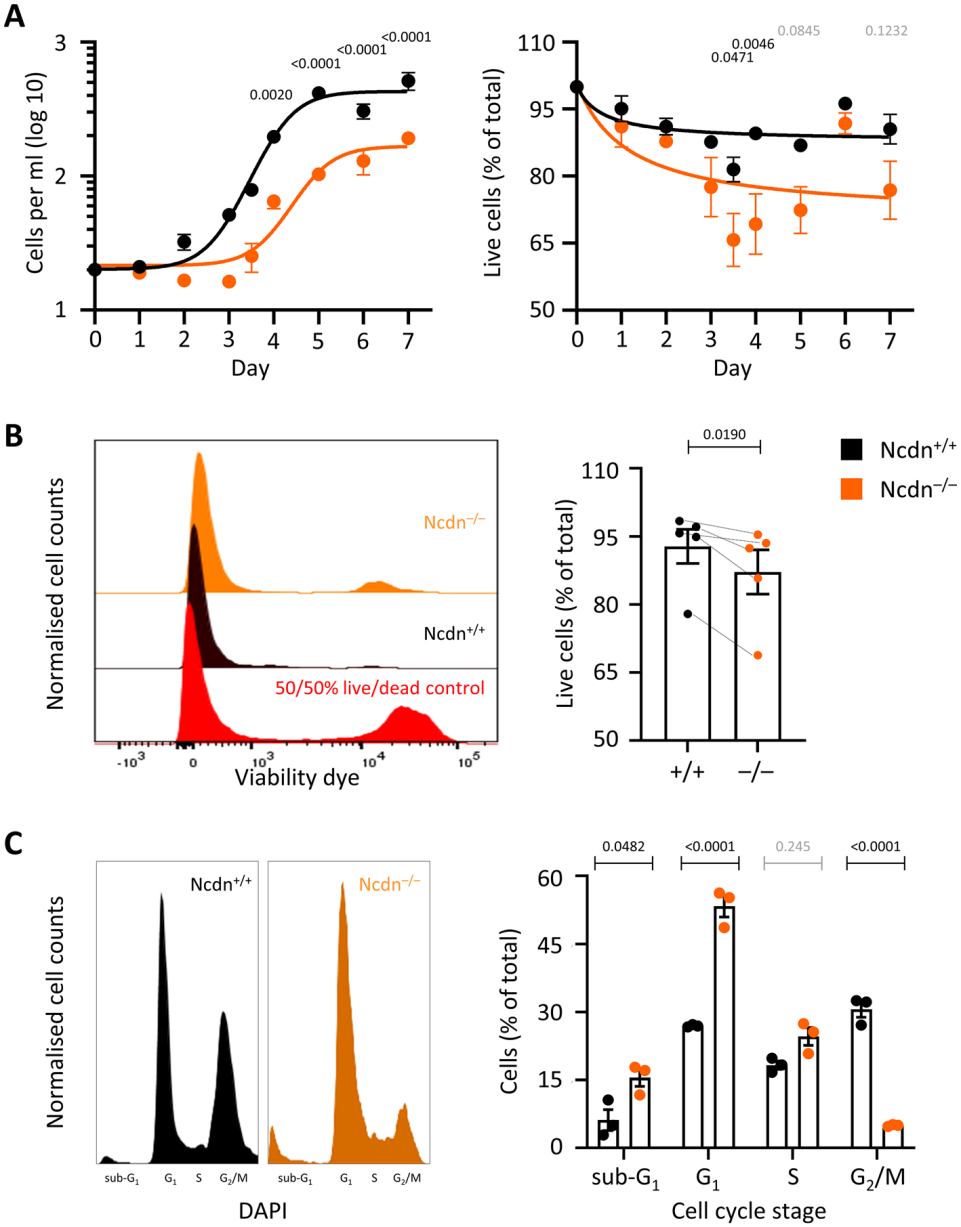
Norbin regulates the growth, viability and cell-cycle progression of PC12-S1PR1 cells**. **(**A**) Cell growth and viability. 20,000 Ncdn^+/+^ (black) and Ncdn^–/–^ (orange) PC12-S1PR1 cells were seeded into a poly-D-lysine coated 12-well plate, and their growth (left-hand panel) and viability (right-hand panel) were assessed over 7 days by haemocytometer with trypan blue exclusion, with one change of medium after three days. Data are mean ± SEM of triplicates from one experiment; the non-linear regressions were fitted using GraphPad. The growth curve is representative of 3 similar experiments. Statistics in are two-way ANOVA with Sidak’s multiple comparisons correction. P-values denote differences between genotypes for a given time point. (**B**) Cell viability. Ncdn^+/+^ (black) and Ncdn^–/–^ (orange) PC12-S1PR1 cells were seeded into 6-well plates, grown to 60% confluence, serum-starved overnight, stained with fixable viability dye and analysed by flow cytometry. Left-hand panel: flow cytometry plots from one experiment representative of five, quantifying the incorporation of fixable viability dye. The red trace shows a control containing 50% heat-killed cells. Right-hand panel: quantification of the proportion of live Ncdn^+/+^ (black) and Ncdn^–/–^ (orange) PC12-S1PR1 cells, as on the left. Data are mean ± SEM of five independent experiments, the same as shown in Fig. 2B. Statistics are paired t-test. (**C**) Cell cycle stages: Ncdn^+/+^ (black) and Ncdn^–/–^ (orange) PC12-S1PR1 cells were grown for two days in a poly-D-lysine coated 6-well plate, fixed, and stained with DAPI prior to analysis of cell cycle stages by flow cytometry. Left-hand panel: flow cytometry plots from one experiment representative of three, quantifying the DAPI signal. Right-hand panel: Quantification of cell cycle stages, as on the left. Data are mean ± SEM of three independent experiments. Statistics are two-way ANOVA with Sidak’s multiple comparisons correction.

Norbin-deficient PC12-S1PR1 cells grew slower than wild type, and their viability appeared reduced, as assessed by haemocytometer and trypan blue dye exclusion (Fig. 3A). Flow cytometric analysis of cell viability, based on the exclusion of a fixable viability dye, confirmed the reduced viability of Norbin-deficient cells (Fig. 3B). We asked if these deficiencies in growth and viability might be caused by an impaired progression through the cell cycle. Indeed, flow cytometric analysis of the cell-cycle stages showed that a greater proportion of Norbin-deficient PC12-S1PR1 cells were in G_1_ phase compared to wild type, and strikingly fewer in G_2_/M (Fig. 3C; see Supplemental Fig. 1A for the gating strategy). The increased cell death was also seen again, as a higher proportion of Norbin-deficient cells were in sub-G_1_ (Fig. 3C). Hence, Norbin is required for the growth, survival and cell-cycle progression of PC12 cells, consistent with the pivotal roles of this protein throughout the nervous system in vivo^4,5,8,9^.

### Norbin regulates the steady-state cell surface level and the agonist-induced internalisation of S1PR1 in PC12 cells

**Figure 4:**
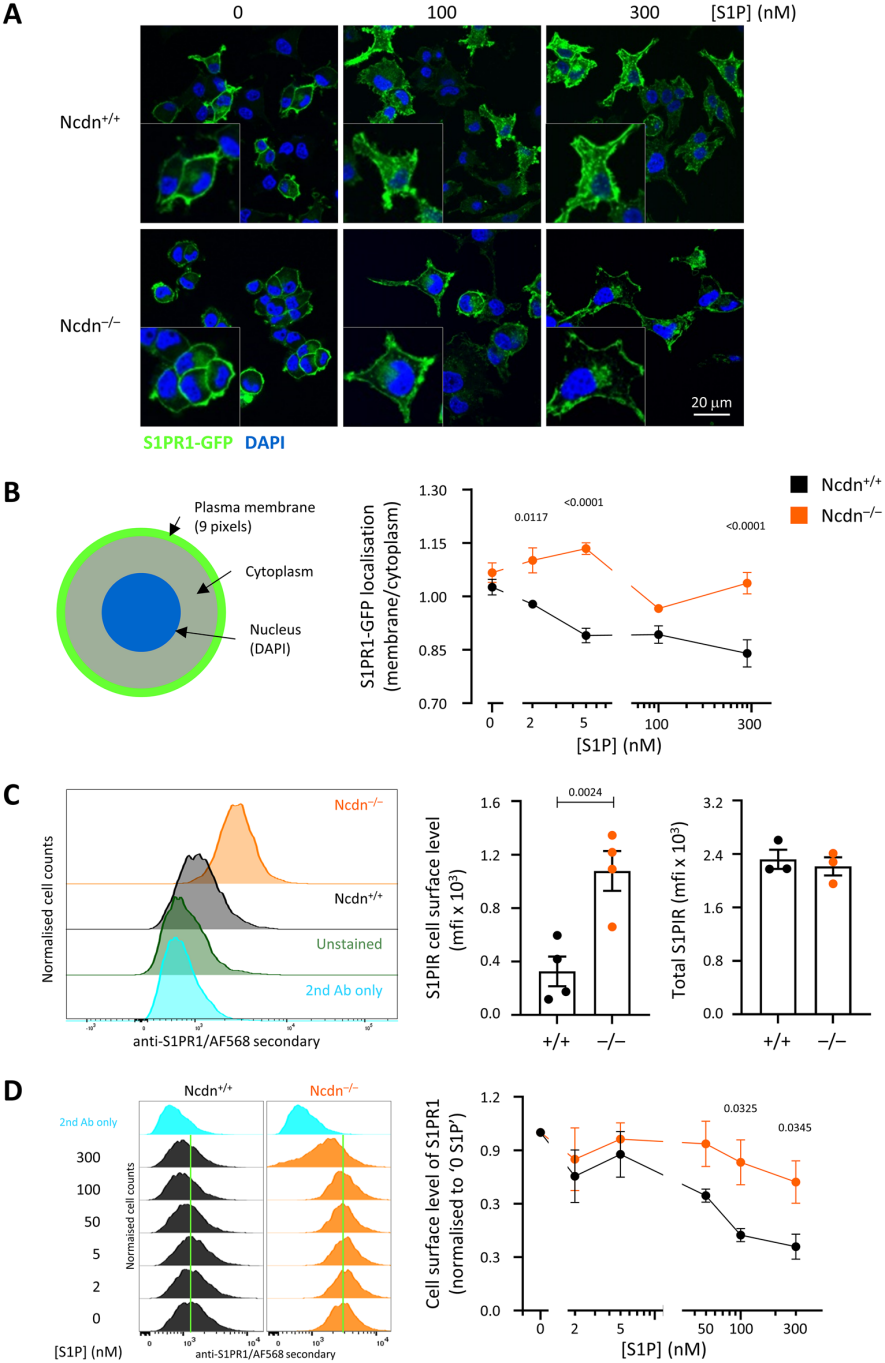
Norbin regulates the steady-state cell-surface level and agonist-induced internalisation of S1PR1 in PC12-S1PR1 cells. (**A**, **B**) Imaging the subcellular localization of S1PR1-GFP. (A) Confocal fluorescence microscopy images of S1PR1-GFP localization in wild type (Ncdn^+/+^) and Norbin-deficient (Ncdn^–/–^) PC12-S1PR1 cells stimulated with the indicated concentrations of S1P for 10 min. Images are from one experiment representative of three. Inserts show 1.76 × magnification. (**B**) Quantification. Confocal images as in (**A**) of cells stimulated with the indicated concentrations of S1P were analysed by CellProfiler for GFP signals in the plasma membrane and cytoplasmic compartments, as illustrated in the schematic. Data are mean ± SEM of triplicate fov from one experiment representative of three; statistics are two-way ANOVA with Sidak’s multiple comparisons correction. P-values denote differences between genotypes for a given S1P concentration. (**C**, **D**) Flow cytometric analysis of the cell surface level of S1PR1. Wild-type (black) and Norbin-deficient (orange) PC12-S1PR1 cells were serum-starved and then mock stimulated (**C**) or stimulated with the indicated doses of S1P (**D**) for 30 min at 37 °C. Cells were stained with S1PR1 primary and AF568-conjugated secondary antibodies, and were analysed by flow cytometry. (**C**) Constitutive cell surface level of S1PR1. Left-hand panel: Flow cytometry plots from one experiment representative of four. Middle panel: Quantification of the constitutive cell surface level of S1PR1 in serum-starved, mock-stimulated cells, as on the left, after taking into account the mean spherical surface area of the cells, calculated from the surface area data in Fig. 2A. Data are mean ± SEM of four independent experiments; statistics are paired t-test. Right-hand panel: Quantification of the total cellular level of S1PR1 was done essentially the same way, except that cells were fixed and permeabilised prior to staining and analysis. Data are mean ± SEM of 3 independent experiments; statistics (paired t-test) showed no difference. (**D**) Left-hand panel: Flow cytometry plots from one experiment representative of four. Right-hand panel: Quantification of the cell surface level of S1PR1 upon stimulation with the indicated concentrations of S1P. Data are mean ± SEM of four independent experiments, normalized to the ‘0 S1P’ condition for both genotypes. Statistics are two-way ANOVA with Sidak’s multiple comparisons correction. *P*-values denote differences between genotypes for a given S1P concentration.

As Norbin is a GPCR adaptor protein that controls GPCR trafficking^5,19,21,22^, and is known to bind the GPCR S1PR1^19^, we investigated if it regulates the subcellular localization of S1PR1-GFP. Confocal microscopy showed that S1PR1-GFP was localized at the plasma membrane of serum-starved, basal cells, as expected^29^, both in wild type and Norbin-deficient PC12-S1PR1 cells (Fig. 4A). Agonist stimulation typically induces the internalisation of GPCRs, including S1PR1, from the plasma membrane into endo-lysosomal compartments, via clathrin-mediated endocytosis, in order to switch off GPCR signalling^29–32^. Confocal microscopy and CellProfiler image analysis showed that S1PR1 was indeed internalised upon stimulation of wild type PC12-S1PR1 cells with S1P, in a dose-dependent manner (*p* = 0.0017) (Fig. 4A–B). In contrast, the receptor remained largely at the plasma membrane in Norbin-deficient PC12-S1PR1 cells (Fig. 4A–B). Therefore, Norbin is required for the agonist-induced internalisation of S1PR1-GFP.

While CellProfiler analysis was a sensitive means of detecting receptor internalisation, it was less suitable for quantifying absolute receptor levels at the cell surface. In addition, PC12-S1PR1 cells express much more endogenous S1PR1 than S1PR1-GFP^28^, and we wished to assess the localisation of both. Therefore, we employed flow cytometry to investigate the cell surface localization of S1PR1 further, using an S1PR1 antibody for detection. The gating strategy is shown in Supplemental Fig. 1B. Under serum-starved, basal conditions, Norbin-deficient PC12-S1PR1 cells had a higher cell surface concentration of S1PR1 than wild type cells, even after taking into account their larger surface area (Fig. 4C). In contrast, the total amount of S1PR1 expressed was normal in Norbin-deficient PC12-S1PR1 cells (Fig. 4C). Hence, Norbin controls the steady-state cell surface level of S1PR1, consistent with previous reports for other GPCRs^5,19,21,22^. Furthermore, similar to the imaging data, the flow cytometry assays showed that S1PR1 was internalized in a dose-dependent manner in wild type PC12-S1PR1 cells upon S1P stimulation (*p* = 0.0124), whereas the receptor remained largely at the plasma membrane in Norbin-deficient cells (Fig. 4D). Overall, these data show that Norbin limits both the constitutive cell surface level of S1PR1 and the agonist-induced internalisation of this receptor. This suggests that Norbin may suppress S1PR1-dependent signalling and responses.

### Norbin limits the S1P-stimulated activation of Akt and p38 Mapk, and is required for the activation of Erk in PC12 cells

**Figure 5:**
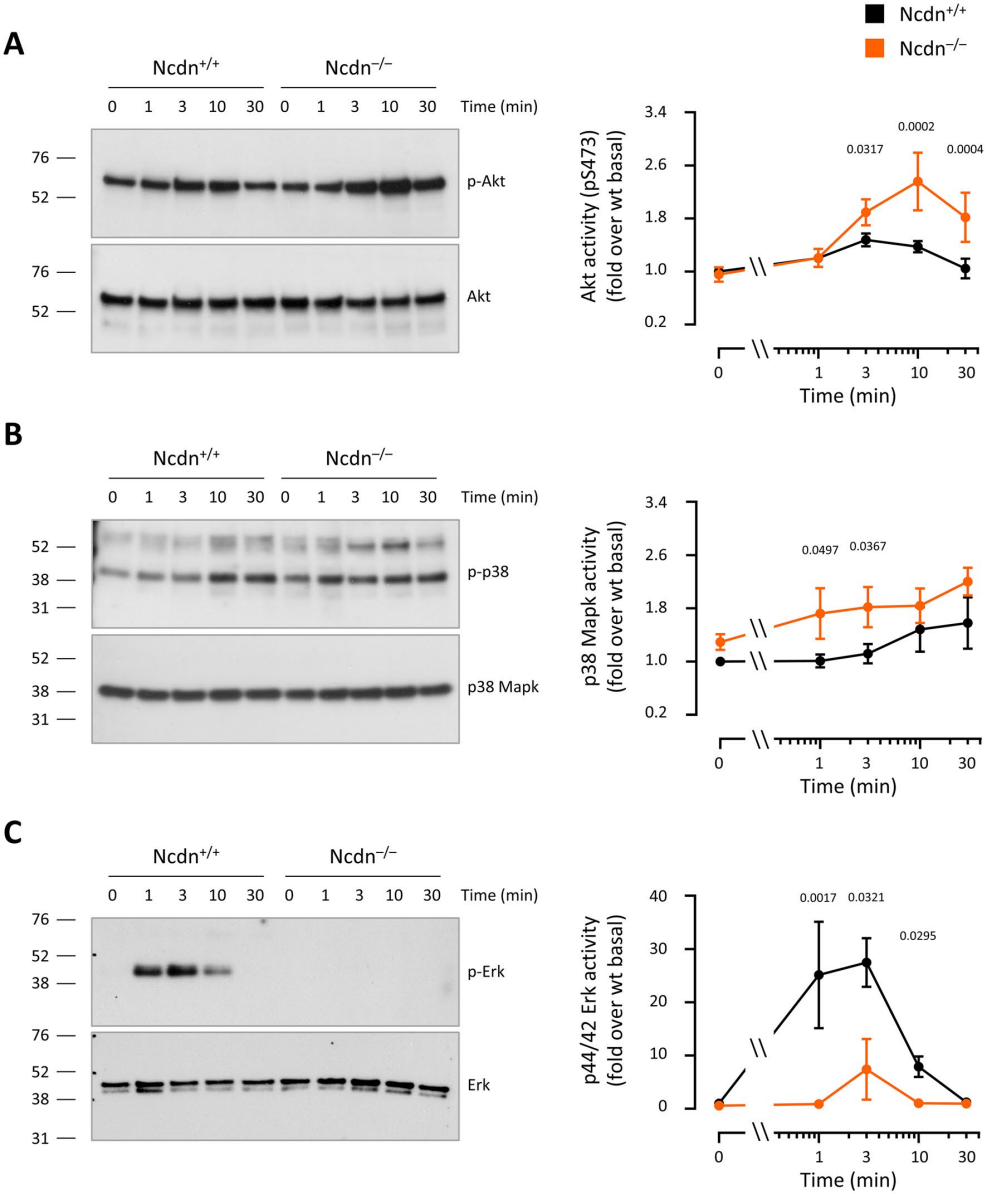
Norbin limits the S1P-stimulated activation of Akt and p38 Mapk, and is required for the activation of Erk in PC12-S1PR1 cells. Wild type (black, Ncdn^+/+^) and Norbin-deficient (orange, Ncdn^–/–^) PC12-S1PR1 cells were serum-starved and then stimulated with 50 nM S1P for the indicated periods of time. Total cell lysates were western blotted for the phosphorylated, active forms of (**A**) Akt, (**B**) p38 Mapk and (**C**) p44/42 Erk. Blots were stripped and reprobed to determine the total amounts of these proteins. Blots shown are from one experiment representative of three. Graphs show the quantification by ImageJ densitometry, normalised to 0’ Ncdn^+/+^. Data are mean ± SEM of 3 independent experiments; statistics are two-way ANOVA with Sidak’s multiple comparisons correction on square-root transformed raw densitometry units. P-values denote differences between genotypes for a given time point.

We previously showed that S1P stimulation increases the activities of Akt and p38 Mapk in wild type PC12-S1PR1 cells^28^. To investigate the effects of Norbin deficiency on S1P signalling, we stimulated serum-starved Norbin-deficient and wild type PC12-S1PR1 with 50 nM S1P for various periods of time up to 30 min, and analysed the activities of Akt, p38 Mapk and p44/42 Erk by phospho-western blotting of total cell lysates. Akt activity (phospho-S473) was stimulated more strongly in the Norbin-deficient cells than wild type cells from 3 min onwards, especially at later time points (Fig. 5A). Hence, Norbin limits the S1P-stimulated activation of Akt. The timing suggest that this might reflect the increased S1PR1 levels at the plasma membrane of the Norbin-deficient cells, due to the reduced agonist-induced receptor internalisation. p38 Mapk activity increased throughout the S1P-stimulation, and was elevated in Norbin-deficient cells compared to wild type, mostly at early timepoints (1 and 3 min) (Fig. 5B). Hence, Norbin limits the S1P-stimulated activation of p38 Mapk, and the timing here suggests that this occurs independently of its effects on S1PR1 trafficking. The S1P-stimulated activation of Erk was robust in wild type PC12-S1PR1 cells, but almost abrogated in the Norbin-deficient cells (Fig. 5C). Therefore, Norbin is required for S1P-stimulated Erk signalling. Overall, Norbin limits the S1P-dependent signalling of Akt and p38 Mapk in PC12 cells, and is required for the activation of Erk. Its effect on Akt activity is likely to be, at least in part, a consequence of its role in S1PR1 trafficking.

## Discussion

Our study shows that the GPCR adaptor protein Norbin controls cell size, morphology, growth, survival and cell-cycle progression, as well as the steady-state cell surface level and agonist-induced internalisation of the GPCR S1PR1, and S1P signalling through Akt, p38 Mapk and Erk, in PC12 cells.

Neuronal functions assigned to Norbin to date are to promote neurite outgrowth and synaptic plasticity^1,2,5,6,17^. In addition, we recently showed that Norbin limits degranulation, phagocytosis, and the production of reactive oxygen species and neutrophil extracellular traps in mouse neutrophils^19^. Here, we showed that Norbin-deficient PC12-S1PR1 cells are constitutively larger and more spread than wild type cells, and their growth, viability, and cell-cycle progression were impaired. The effect on cell spreading was not entirely unexpected, as we previously showed increased spreading in Norbin-deficient neutrophils^19^. This may be a consequence of Norbin regulating the actin cytoskeleton, possibly through its interaction with actin cytoskeleton regulating protein mDia^33^, and through its regulation of the activities of Rac small GTPases and the Rac guanine-nucleotide exchange factors Prex1 and Vav^18,19^. Interestingly, we recently found that Prex1 deficiency has similar consequences for the morphology, growth and cell-cycle progression of PC12-S1PR1 cells to Norbin deficiency^28^, suggesting that Prex1 may be a Norbin target in neurons. We did not observe any altered neurite formation in Norbin-deficient PC12-S1PR1 cells, as the circularity of these cells was normal. However, we only investigated the morphology of serum-starved, undifferentiated PC12 cells. Stimulation with S1P, or other agonists that induce protrusions, or differentiation of the cells with NGF, may have revealed further effects on cell morphology. For example, we would expect to see reduced neurite formation in differentiated Norbin-deficient PC12 cells, as was previously reported upon Norbin knock-down^17^. Roles of Norbin in cell growth, viability and cell-cycle progression have, to our knowledge, not been described before. However, these could easily have been missed by previous studies on cellular Norbin functions, because these studies relied largely on overexpression^2,5,8,18,20,21^, or on work with acutely isolated primary mouse cells^5,8,19^, rather than deletion of Norbin in a cultured cell line that allowed longitudinal observations. The effects we observed were striking and may explain the essential requirement for Norbin in early embryonic development^4^. The mechanisms underlying the effects of Norbin on these key cell responses remain to be explored.

We saw an increased constitutive surface level of S1PR1 in Norbin-deficient PC12 cells. This was perhaps unsurprising, as Norbin is known to bind S1PR1^20^, and its regulation of the steady-state surface levels of other GPCRs (mGluR5, MCHR1, C5aR1, CXCR4) was previously reported^5,19,21,22^. However, it was not predictable in which way Norbin would affect S1PR1 levels. Norbin deficiency caused constitutively increased cell surface levels of C5aR1 and CXCR4 in neutrophils^19^, as it did here with S1PR1, whereas it reduced the surface level of mGluR5 in cortical and hippocampal neurons^5,22^. Hence, Norbin seems to have receptor- and/or cell type-specific effects on steady-state GPCR trafficking. Like most GPCRs, S1PR1 is internalised upon agonist stimulation by clathrin-mediated endocytosis, which serves to attenuate S1PR1 signalling^29,31,32^. Here, we demonstrated impaired agonist-induced internalisation of S1PR1 in Norbin-deficient PC12 cells. This was again not predictable, as Norbin was previously shown not to affect the agonist-induced internalisation of MCHR1^21^, whereas it was required for the agonist-induced internalisation of mGluR5^22^. Our findings suggest that Norbin plays a wider role in the homologous desensitization of GPCRs than previously appreciated.

The mechanisms through which Norbin regulates GPCR trafficking remain unclear, although it is certain that direct binding between Norbin and the GPCR is required^5,22^. It seems possible that Norbin binding may prevent the interaction of the GPCR with receptor kinases or β-arrestin. In addition to trafficking, Norbin also controls GPCR signalling, either positively or negatively. For example, coexpression of Norbin with MHCR1 inhibited MCH-dependent Ca^2+^ signalling in HEK293 cells^21^, whereas coexpression with mGluR5 increased dihydroxy-phenylglycine-induced Ca^2+^ signalling^5^. We previously described increased Erk and Rac signalling in Norbin-deficient neutrophils stimulated with the FPR1 ligand fMLP^19^. Here, we demonstrated that Norbin limits the S1P-stimulated activation of Akt and p38 Mapk in PC12 cells, and was required for Erk activation under the same conditions. This is, to our knowledge, the first report on Norbin regulating p38 Mapk activity. Interestingly, Erk activation was robust in wild type cells and abrogated in Norbin-deficient cells when assayed with an antibody that recognises phospho-Thr202 alone or in combination with phospho-Tyr204 (Cell Signaling Technologies 4370). In contrast, it was barely detectable and identical between the genotypes when we used an antibody that requires phosphorylation of both residues (Cell Signaling Technologies 9102; data not shown). Phosphorylation of either residue by the dual-specificity kinase Mek results in partial Erk activation^34,35^, and it appears that Norbin is indirectly required for the phosphorylation of phospho-Thr202 at least.

It remains largely unclear where Norbin affects GPCR signalling merely through its role in GPCR trafficking, and where it controls additional aspects. There are, however, several examples where Norbin effects on GPCR signalling are too fast to be trafficking-related, for instance in the fMLP-stimulated activation of Rac in neutrophils^19^, or where they are seen although GPCR trafficking appears unaffected, such as in Ca^2+^ signalling downstream of MCHR1^21^. This appears to depend on the GPCR, cellular context, and signalling pathway, and requires further investigation in the future. The kinetics of the signalling responses tested here suggest that Norbin may regulate p38 and Erk independently of GPCR trafficking, whereas its effects on Akt activity may be a consequence of the altered agonist-induced S1PR1 internalisation.

Norbin is an adaptor protein without enzymatic activity that functions by binding GPCRs and its effector proteins P-Rex1 and PKA, which signal downstream of GPCRs^1^. The molecular mechanisms through which Norbin exerts its adaptor roles require more research in the future. In particular, a search for further effector proteins such as those through which Norbin regulates GPCR trafficking will be required. Furthermore, it remains unknown whether Norbin is governed by any form of regulation. The protein has no discernible domains, and its interaction with GPCRs is constitutive. However, there is some evidence for autoinhibition, as the isolated N-terminus has greater neurite outgrowth-promoting activity than full-length Norbin^33^. It would be interesting to search for signals that relieve this putative autoinhibition and evaluate their consequences for GPCR trafficking, signalling and cell responses.

In summary, our report identified novel functional roles of Norbin in PC12 cells, controlling the size, morphology, growth, survival, and cell cycle, plus we consolidated the recent finding that Norbin cannot only control the steady-state surface levels of GPCRs but also their agonist-induced internalisation^22^. We did not seek to investigate here any links between cell responses and S1PR1 trafficking/signalling, because the former were observed in serum-starved, basal cells, without the addition of S1P, whereas the signalling was only affected in the presence of S1P. It is, however, possible to draw some parallels. Erk is the archetypical regulator of cell cycle progression and proliferation^36^, and Erk activity, cell cycle progression and proliferation were all impaired in Norbin-deficient cells, suggesting that Norbin may regulate these cell responses through Erk. Akt regulates cell size^37^, so the elevated Akt activity might underlie the increased size of Norbin-deficient cells. Similarly, p38 Mapk activity can induce cell death under some conditions^38^, and may be involved in this process here. Yet, the correlations between signalling and cell responses are not clear-cut, as Akt activity is generally associated with cell survival^37^, the opposite of what we observed. Altogether, is conceivable that the effects of Norbin on GPCR trafficking/signalling and cell responses are linked. Like many cell types, PC12 cells may produce S1P in an autocrine/paracrine fashion^39^, and the increased steady-state surface level of S1PR1 in Norbin-deficient cells might result in chronically altered S1PR1 cell signalling and responses, as S1PR1 is known to regulate the morphology, proliferation and viability of various cell types^40,41^. Approaches such as careful titration of receptor antagonists or the use of Norbin mutants that cannot bind GPCRs, like the one recently reported by Ojha et al. to block the interaction with mGluR5^22^, may prove useful in the future in determining the molecular mechanisms that link Norbin-dependent GPCR trafficking and signalling to its control of cell responses.

### Supplementary Information

Supplementary Information.

## Data Availability

The sequencing dataset of the Norbin-deficient PC12-S1PR1 cell clone generated and analysed during the current study is available in the GenBank repository, accession number OQ559938. All other data are contained within the article.
