# Supplementary material for: The GPCR adaptor protein Norbin regulates S1PR1 trafficking and the morphology, cell cycle and survival of PC12 cells
Source: Sci Rep. 2023 Oct 25;13:18237. doi: 10.1038/s41598-023-45148-6 (PMC10600135; doi:10.1038/s41598-023-45148-6)

Supplemental Material for

The GPCR Adaptor Protein Norbin Regulates S1PR1 Trafficking, and the Morphology, Cell Cycle and Survival of PC12 Cells

Valdemar B. I. Johansen [1,2,3], Elizabeth Hampson [1,4], Elpida Tsonou [1,5], Chiara Pantarelli [1], Julia Y. Chu [1], Laraine Crossland [1], Hanneke Okkenhaug [6], Andrew J. Massey [4], David C. Hornigold [5], Heidi C. E. Welch [1,7], Stephen A. Chetwynd [1,7]

Supplemental Figure 1

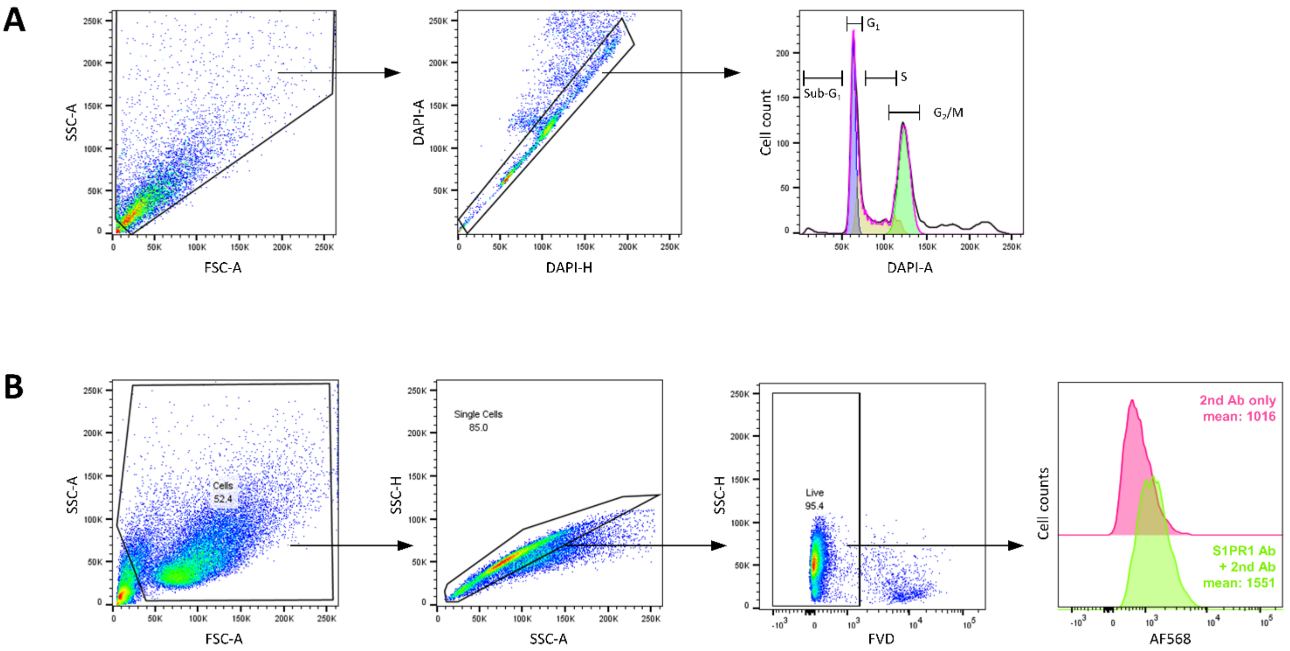

Supplemental Figure 1. Flow cytometry gating strategies. (A) Gating strategy for Figure 3C, cell-cycle analysis. Representative flow cytometry plots showing gates to exclude cell debris (left-hand panel) and any non-single cells (middle panel). Single cells were examined for DNA-content by their level of DAPI incorporation (right-hand panel). **(B)** Gating strategy for Figures 2B, 3B, 4C and 4D. After gating to exclude debris (left-hand panel) and any non-single cells (second panel), fixable viability dye was used to identify dead cells (third panel). Live cells were then analysed further for the cell surface level of S1PR1 (right-hand panel), identified by staining with S1PR1 antibody and AF658-conjugated secondary antibody (green), compared to cells incubated with secondary antibody only (pink).

Uncropped gels and blots:

for Figure 1B:

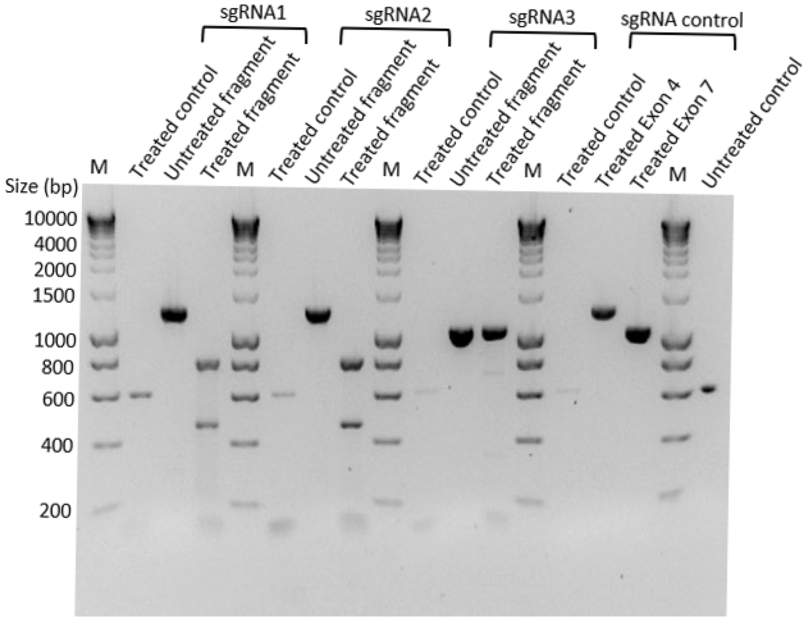

for Figure 1D:

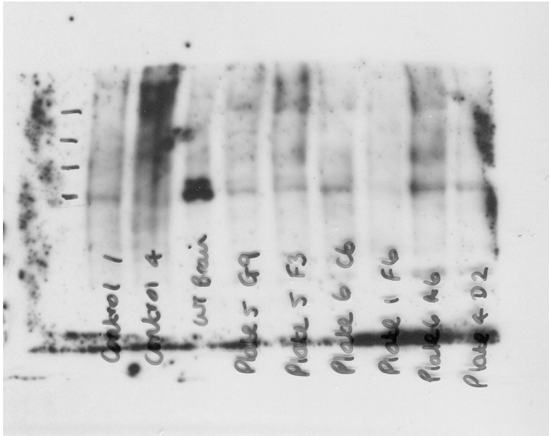

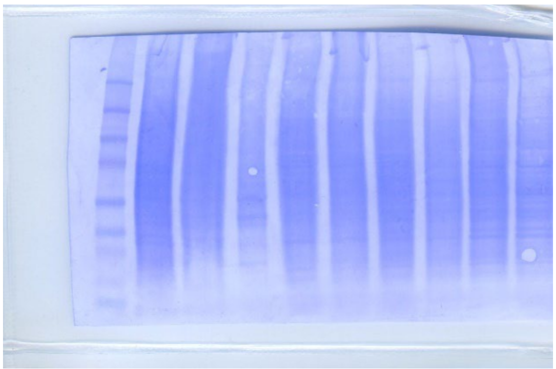

for Figure 5A:

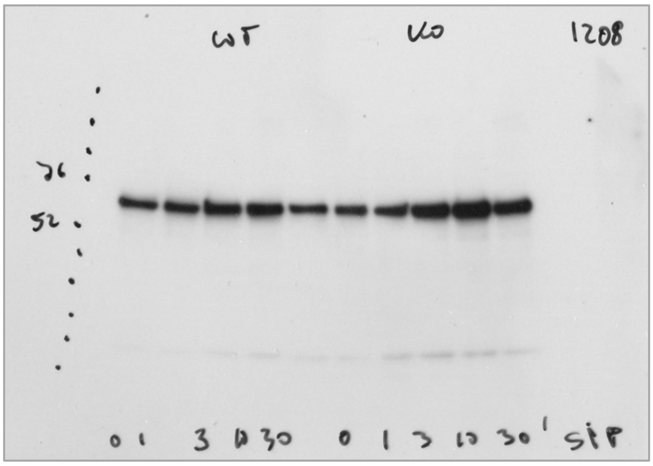

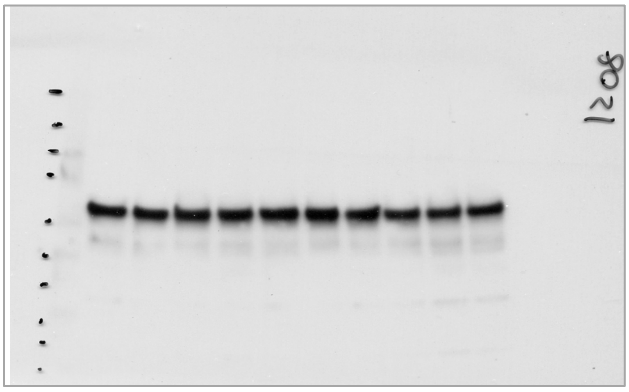

for Figure 5B:

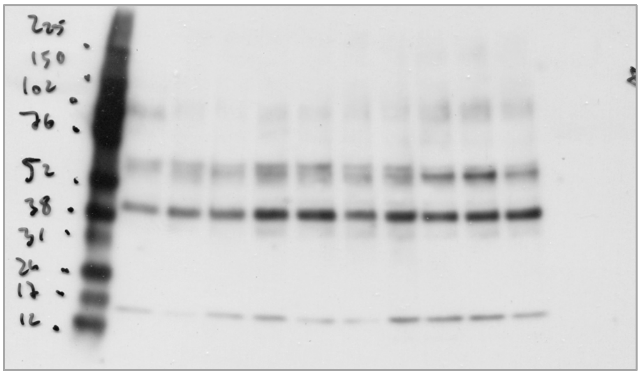

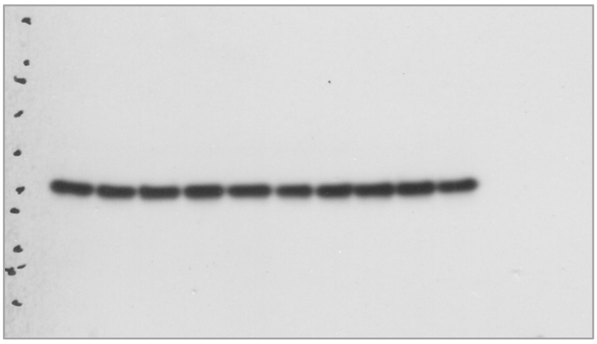

for Figure 5C:

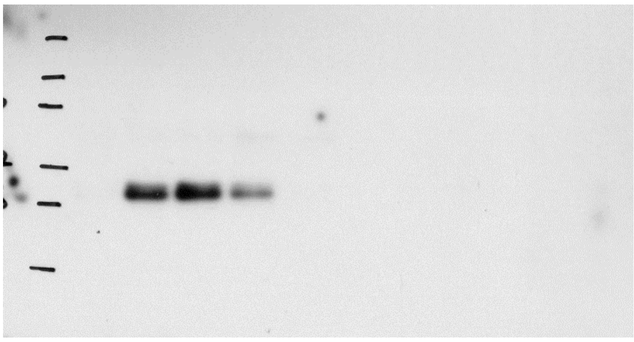

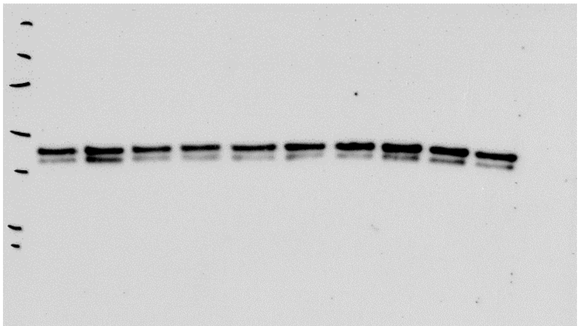

Supplement: Supplementary file 1 — Supplementary Information. [file 41598_2023_45148_MOESM1_ESM.pdf]
